# Stress hormone concentration in Rocky Mountain populations of the American pika (*Ochotona princeps*)

**DOI:** 10.1093/conphys/cot027

**Published:** 2013-10-18

**Authors:** Jennifer L. Wilkening, Chris Ray, Karen L. Sweazea

**Affiliations:** 1 Department of Ecology and Evolutionary Biology, University of Colorado, Boulder, CO 80309-0334, USA; 2 School of Nutrition and Health Promotion and School of Life Sciences, Arizona State University, Tempe, AZ 85287, USA

**Keywords:** Climate change, field endocrinology, sentinel alpine species, stress

## Abstract

The American pika (*Ochotona princeps*) is considered a sentinel species for detecting ecological effects of climate change. Pikas are declining within a large portion of their range, but previous studies have focused only on local pika extinction as a metric of change. We designed a procedure which can provide an earlier warning signal, based on non-invasive sampling and analysis of physiological stress in living pikas. Pikas were sampled at several locations in the Rocky Mountains for the measurement of glucocorticoid metabolites (GCMs) in faeces. Using a time series of faecal pellets from 12 individuals, we detected a significant increase in faecal GCM level in response to capture, thus biologically validating the use of a corticosterone enzyme immunoassay. We also established baseline, peak, and post-peak GCM concentrations for pikas in the Rocky Mountains, which varied according to gender and individual. This is the first study to measure stress hormone metabolites in any species of pika. The methods developed and validated in this study can be used to add non-invasive measurements of physiological stress to pika monitoring programmes and other research designed to assess pika vulnerability to predicted changes in climate. Pika monitoring programmes currently in place use a protocol that relates current site use by pikas with data on local habitat characteristics, such as elevation, to infer potential effects of climate change. Data generated by these monitoring studies can be used to identify the trends in site use by pikas in relationship to habitat covariates. However, this approach does not take into account the role of behavioural thermoregulation and the pika's use of microhabitats to ameliorate variations in climate. Incorporating a stress metric, such as GCM concentration, will provide relatively direct evidence for or against the hypothesis that pikas can be stressed by climate regardless of behavioural adaptations.

## Abstract (precis)

We developed and validated techniques for non-invasive measurement of physiological stress in the American pika, a climate sensitive sentinel species. Results deomnstrate baseline stress hormone levels for pikas, and establish a basis for future research to determine whether local habitat variables specifically related to climate can explain levels of stress.

## Introduction

Due to the severity of change in alpine climates, alpine mammals are predicted to be among the species most threatened by climate change (Hughes, 2000; Parmesan, 2006; Moritz *et al.*, 2008). The American pika (*Ochotona princeps*), which occurs only where it can access cool microclimates, is considered a sentinel species for detecting ecological effects of climate change (McDonald and Brown, 1992; Hafner, 1993, 1994; Lawlor, 1998; Beever *et al.*, 2003; Krajick, 2004; Smith *et al.*, 2004; Grayson, 2005; Morrison and Hik, 2007). The lowest recorded occurrence of pikas in the Great Basin has risen 150 m during the past century (Grayson, 2005), and climate has been implicated as a driver of recent pika losses in the Great Basin (Beever *et al.*, 2010, 2011; Wilkening *et al.*, 2011). Projections suggest that pikas in the USA may disappear from 55% (95% confidence interval = 35–66%) of their current range even under low-emission scenarios (Ray *et al.*, 2012), and the species has been considered for listing as threatened or endangered at both federal and state levels (USFWS, 2010; Osborn and Applebee, 2011). The pika's recent, apparently climate-driven range retraction has been highly publicized, and there has been a growing interest in documenting changes in pika distribution. Research efforts have intensified over the past decade, and government agencies and citizen science organizations throughout the western USA have initiated programmes to monitor pika distribution and assess vulnerability to predicted climate change (Garrett *et al.*, 2011).

Studies of the American pika are well suited for testing hypotheses regarding the potential nature of climate-mediated range shifts. This species has a narrow thermal tolerance, owing to its relatively high metabolic rate and low thermal conductance (MacArthur and Wang, 1973). Pikas do not hibernate, and most pika species rely on vegetation stored in the form of ‘haypiles’ to supply nutritional needs throughout the winter (Dearing, 1997). Collecting this vegetation during the summer is a highly energetic activity, generating body heat that must be dissipated passively (MacArthur and Wang, 1974; Smith, 1974). Consequently, this species is a habitat specialist, occurring in the western USA only in taluses, boulder fields, lava beds, and similar areas of blocky debris which traps cooler air in pockets between the rocks (Smith and Weston, 1990; Millar and Westfall, 2010).

Most efforts to understand effects of climate change on the pika and other species have relied on establishing relationships between habitat characteristics (such as temperature and elevation) and the distribution of the species (e.g. Beever *et al.*, 2003, 2010; Grayson, 2005). The major determinants of range for many species are temperature and precipitation, and these two factors also appear to control recent pika dynamics. Several studies have shown that the American pika is more likely to occur and persist at sites with higher precipitation, where surface and sub-surface temperatures are less extreme, and where vegetation communities are dominated by forbs (Ray and Beever, 2007; Beever *et al.*, 2010, 2011; Millar and Westfall, 2010; Rodhouse *et al.*, 2010; Erb *et al.*, 2011; Wilkening *et al.*, 2011). These results suggest both direct and indirect effects of temperature and precipitation on the distribution of the species. Identifying more precisely which habitat characteristics are associated with individual pika fitness will improve our understanding of potential effects of climate change. Without evidence of a direct climatic impact on individual pikas, studies correlating pika presence with climate metrics provide relatively weak evidence for projecting effects of climate change on this species. For a species that thermoregulates behaviourally by switching between surface and sub-surface habitats, it may be especially difficult to use presence/absence data to identify the specific habitat or climatic variables driving range dynamics.

A more direct method for identifying potential climatic stressors is to measure physiological stress in individuals. This approach has the added benefit of being able to identify stressors before local extinctions have occurred. It can also suggest which factors stress individuals directly vs. indirectly; for example, individual stress metrics might be lagged with respect to precipitation, suggesting indirect effects mediated by vegetation quality. Measuring physiological stress may be preferable to other metrics of fitness that require mark–recapture studies, which can be disruptive to the animal and resource intensive.

One stress metric that can be measured non-invasively is glucocorticoid (GC) concentration. In response to a stressor, vertebrates release glucocorticoid hormones (such as cortisol or corticosterone), which can then be measured in physiological samples (such as plasma, saliva, or faeces). This release of GCs in response to short-term stress permits rapid energy mobilization (Sapolsky *et al.*, 2000) and behavioural changes, which can result in improved survival and fitness (Bonier *et al.*, 2009). An evolved stress response is often what enables vertebrates to respond successfully to daily or seasonal environmental variability (Romero, 2002). However, continued release of GCs resulting from chronic stress can lead to potentially harmful effects, such as the death of nerve cells, hyperglycaemia, muscle and bone atrophy, hypertension, immunosuppression, and reduced reproduction (Riley, 1981; McEwen and Sapolsky, 1995; von Holst, 1998; Wingfield and Sapolsky, 2003; Engelmann *et al.*, 2004; Romero, 2004; Boonstra, 2005; Korte *et al.*, 2005).

Glucocorticoids travel to their target tissues via the blood, and the concentration of GC in plasma has traditionally been used to assess stress in various species (Wingfield *et al.*, 1992, 1994; von Holst, 1998; Sapolsky *et al.*, 2000; Mostl and Palme, 2002; Romero, 2002, 2004; Wingfield and Sapolsky, 2003; Korte *et al.*, 2005). However, animals must be captured in order to collect blood, and circulating hormone levels can increase rapidly in response to capture and handling stress (Cook *et al.*, 2000; Romero, 2004). Samples collected after 3 min of capture are not considered to be baseline measurements, and for many wild animals, blood samples can rarely be obtained within this time frame using common trapping techniques. Furthermore, the capture and handling of rare or endangered species may not be possible, which underscores the importance of developing alternative methods of GC measurement.

Non-invasive methods to quantify stress have been developed for many different taxa. The measurement of glucocorticoid metabolites (GCMs) found in faecal samples is now one of the most common of these methods (Miller *et al.*, 1991; Kirkpatrick *et al.*, 1996; Palme *et al.*, 1996; Berkeley *et al.*, 1997; Jurke *et al.*, 1997; Wasser *et al.*, 1997; Goymann *et al.*, 1999; Harper and Austad, 2000; Dehnhard *et al.*, 2001; Ganswindt *et al.*, 2003; Turner *et al.*, 2003; Hunt *et al.*, 2004; Touma *et al.*, 2004; Cyr and Romero, 2008; Chinnadurai *et al.*, 2009; Sheriff *et al.*, 2009; Ashley *et al.*, 2011; Scarlata *et al.*, 2011; Hogan *et al.*, 2012; Howell-Stephens *et al.*, 2012; Laver *et al.*, 2012; Narayan *et al.*, 2012, 2013; Shutt *et al.*, 2012; Smith *et al.*, 2012). Steroid hormones circulating in the body are metabolized by the liver, then excreted as metabolites into the gut (Taylor, 1971; Palme *et al.*, 1996; Mostl and Palme, 2002), and GCMs can be detected in the excrement of birds and mammals. This technique offers several advantages. In many cases, faecal samples can be collected relatively easily without disturbing or endangering the animal, which allows for repeated sampling over time (Mostl and Palme, 2002; Millspaugh and Washburn, 2004). Additionally, GCM measurements obtained from collected faecal samples are not biased by capture-induced stress, and may provide a more precise assessment of the endocrine condition of an animal (Harper and Austad, 2000; Millspaugh *et al.*, 2001; Touma *et al.*, 2003). Finally, GCM measurements typically reflect an average level of circulating glucocorticoids over time. Unlike a blood sample taken at a single point in time, GCM concentrations appear to be less affected by the pulsatile nature of hormone secretion (Palme *et al.*, 1996; Harper and Austad, 2000; Palme, 2005); therefore, GCM concentrations may represent the average stress level of an animal more accurately than a plasma sample (Touma and Palme, 2005).

Here we present the first study to develop and validate methods that can be used non-invasively to measure physiological stress in pikas. Unlike other members of the family Ochotonidae, American pikas are easily stressed in captivity and experience high mortality in laboratory settings (Krear, 1965; MacArthur and Wang, 1974). Several zoos have been unsuccessful in their attempts to maintain viable populations of pikas (http://www.zoochat.com/2/pikas-zoos-301266/; accessed 5/7/13), and there are currently no captive populations of American pikas in zoos or laboratory settings. Non-invasive methods of assessing stress are particularly useful for studying the stress response of a species sensitive to variation in its natural habitat.

The first objective of our study was to validate an enzyme immunoassay biologically for measuring GCMs in pika faeces. Although GCM measurement has been used for over 20  years in numerous wildlife species, GCMs have never been quantified for any species of pika. When applying this technique to a species for the first time, validation of all protocols related to sample storage, extraction procedure, and assay selection must occur in order to enable reliable interpretation of results (Touma and Palme, 2005). We collected a time series of faecal pellets from individual pikas that were live trapped and later released, to measure individual baseline GCM levels followed by the faecal GCM response to capture.

Our second objective was to investigate individual and gender-based differences in GCM concentration.

Our third objective was to establish baseline GCM measurements for pikas, which could be used as a basis for interpreting GCM levels measured in future samples. At the level of individuals, we used our time series to establish pre-capture (pre-stress) GCM levels. At the level of populations, we compared these baseline stress metrics between pikas occurring at different latitudes, to investigate environmental trends. We focused on pika populations occurring in the Rocky Mountains, where this species has not experienced the magnitude of decline observed in the Great Basin (Erb *et al.*, 2011), and may better represent baseline physiology for the species.

Finally, we present several conservation applications, and discuss how our established techniques can be used in conjunction with ongoing pika research to develop a greater understanding of climate change impacts for this indicator species.

## Materials and methods

### Study area and animal sampling

Pikas were non-lethally sampled in the Rocky Mountains in July-August 2011–2012 at Niwot Ridge long-term ecological research site (LTER) and Brainard Lake Recreation Area, both located in Boulder County, (CO, USA), and Emerald Lake, located in Gallatin County (MT, USA). Study sites were located in areas of typical pika habitat, characterized by large regions of broken rock (talus) interspersed with alpine meadows ranging in elevation from 2500 to 3700 m. Pikas were captured using wire-mesh traps (Tomahawk model 201) placed near pika haypiles and provisioned with local vegetation (forbs and grasses native to the Rocky Mountains supplemented with commercial salad greens). Traps were checked within 2 h of setting them. Prior to handling, animals were self-transferred to an induction chamber containing an inhalant anaesthetic agent (isoflurane). Light anaesthesia was maintained throughout the 20 min handling process, using repeated induction if necessary. Each summer, ∼50 pikas were sampled and ear-tagged for a long-term study of individual survival. Demographic data (stage and sex) and physiological samples (faeces) were collected at point of capture, and most animals were released immediately. Eight adult males, one juvenile male, six adult females, and one juvenile female were held on site for ∼24 h in a holding chamber specifically designed for collection of faecal samples. The holding chamber consisted of a plastic bin (length× width × height = 60 cm × 30 cm × 16 cm) with a detachable lid, modified to include an internal air thermometer, air vents, and a mesh screen floor with pull-out drawer for periodic faecal sample removal. The chamber was provisioned with surplus vegetation (as described for traps) pre-moistened with water using a mister. Individuals were retained inside the chamber one at a time, and researchers monitored the holding chamber continually. Each pika was released at point of capture at the end of the holding period. Trapping and sampling procedures were reviewed and authorized by Colorado Parks and Wildlife (license no. TR2014) and the University of Colorado-Boulder Institutional Animal Care and Use Committee (protocol 1104.06).

Like all lagomorphs, pikas are coprophagic and excrete two different types of faeces. Caecal faeces contain partly digested vegetation and are often re-ingested in order to obtain additional nutrients. Faecal pellets, which are not re-ingested, are the more common type of faecal material found in natural environments. Given that non-invasive studies of pika stress hormone levels will be likely to involve this more abundant type of faeces, only faecal pellets were analysed for this study. In order to ensure further that our faecal samples were similar to what would be available for non-invasive studies, we analysed only faecal pellets that had been urinated on. Pikas maintain established latrines in the wild, and habitually urinate on their faecal droppings. Pikas in the holding chamber were watched constantly, and researchers collected most faecal samples within minutes of excretion. For the majority of individuals, faecal samples were collected regularly every 2–4 h; however, some animals (*n* = 3) did not defaecate until 6–8 h after the time of capture. The time of sample collection (0–23 h) was recorded for each sample. Faecal samples were held on ice in the field, and were transferred within 48 h to a −20°C freezer at the University of Colorado, Boulder (CO, USA).

### Extraction of hormone metabolites and enzyme immunoassays

Each sample was lyophilized and ground into powder using a mortar and pestle, then subjected to the steroid solid extraction protocol given by Arbor Assay Design, Inc. (Ann Arbor, MI, USA). Briefly, 0.100 g of dried faecal material was blended with 1.000 ml of 90% aqueous ethanol using a vortex shaker (Vortex Genie 2; Scientific Industries, Inc.). Samples were shaken for 1 h at 350 r.p.m. at room temperature to ensure thorough extraction of the hormone metabolite. They were then centrifuged at 5000 r.p.m. for 15  min, and the resulting supernatant was drawn off and transferred to a clean tube for evaporation. The supernatant solution was evaporated to dryness in a vacuum centrifuge, and extracts were stored at −20°C. Immediately prior to enzyme immunoassay analysis, extracts were reconstituted with a mixture of ethanol and assay buffer (1:20 ratio v/v) and were mixed completely using the above vortex shaker.

**Figure 1.**
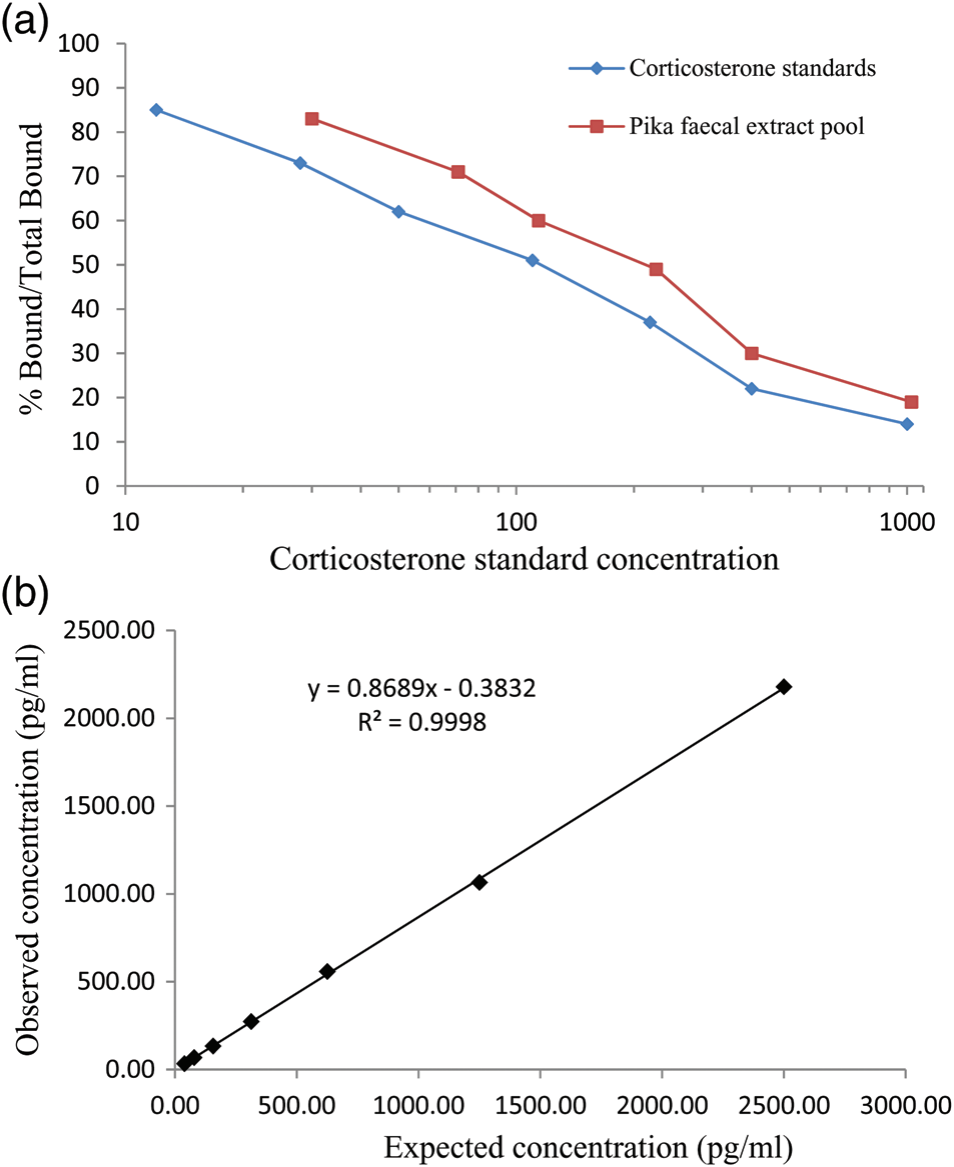
Binding displacement curves of serially diluted pika faecal extracts (**a**) and recovery of exogenous corticosterone standard added to pika faecal extracts (**b**).

Comparative analysis of GCM levels in samples was conducted using a commercially available corticosterone enzyme immunoassay kit (Arbor Assay Design, Inc.; catalogue no. K014-H1). The enzyme immunoassay used a sheep polyclonal antibody specific for corticosterone, and was designed to measure total corticosterone in numerous physiological samples, including extracted faecal samples. Corticosterone, rather than cortisol, was measured in pikas because corticosterone was found to be the major GC in other lagomorphs (Teskey-Gerstl *et al.*, 2000; Monclus *et al.*, 2006; Rehnus *et al.*, 2009; Sheriff *et al.*, 2009; Scarlata *et al.*, 2011). During each assay, we ran extracted samples in triplicate along with seven known concentrations of corticosterone (5000, 2500, 1250, 625, 312.50, 156.25, and 78.125 pg/ml) A standard curve was constructed using results from known corticosterone concentrations. Values for each extracted sample were generated using a microplate reader (BioTek Microplate Reader Synergy HT; 2005 Biotek Industries, Inc.) and Gen 5 1.11 Data Analysis software. Intra-assay coefficients of variation were 5.2 and 3.1% for the low and high binding controls, and inter-assay coefficients of variation were 12.6 and 11.4%, respectively. Final concentrations of faecal GCM were expressed as nanograms per gram of dry faeces. To validate the use of the corticosterone enzyme immunoassay kit, we demonstrated the following characteristics: (i) parallelism between the standard curve and serial dilutions of pooled faecal samples; and (ii) significant recovery (86%) of corticosterone standard added to faecal extracts prior to analysis (Fig. 1a and b; Touma and Palme, 2005). The appropriate dilution (1:20) was calculated from the parallelism results.

### Data analysis

Data were analysed separately by sex (male and female) and age (adult and juvenile). Given that faecal sample production was inconstant over time, we binned samples by time elapsed since capture to characterize the timing of any GCM response better. Samples were binned into six periods of 5 h each (0:00–5:00, 5:01–10:00, 10:01–15:00, 15:01–20:00, 20:01–25:00, and 25:01–30:00 h:min after capture). Glucocorticoid metabolite levels were averaged within time periods for each individual. Thus, the timing of ‘peak’ (highest) GCM level for each individual was determined to within 2.5 h. Pre-peak ‘baseline’ GCM level was calculated for each individual by averaging all samples within all time periods prior to the individual's peak GCM level. Likewise, post-peak GCM level was calculated by averaging all samples within all time periods after the individual's peak GCM level. One-way ANOVA was used to test for the following attributes: (i) differences among individuals in baseline GCM levels; (ii) differences between males and females in baseline GCM levels; and (iii) differences over time in post-stress GCM levels. Normal probability plots were examined, and a Shapiro–Wilk statistic was calculated to test for normality. Tukey's Honest Significant Difference test was used to identify differences between peak and non-peak GCM levels for adult pikas. All statistical analyses were conducted using R 3.0.1 (R Core Team, 2013), and significance was assessed at the α = 0.05 level.

## Results

### Characterization of baseline, peak, and post-peak glucocorticoid metabolite levels

**Figure 2.**
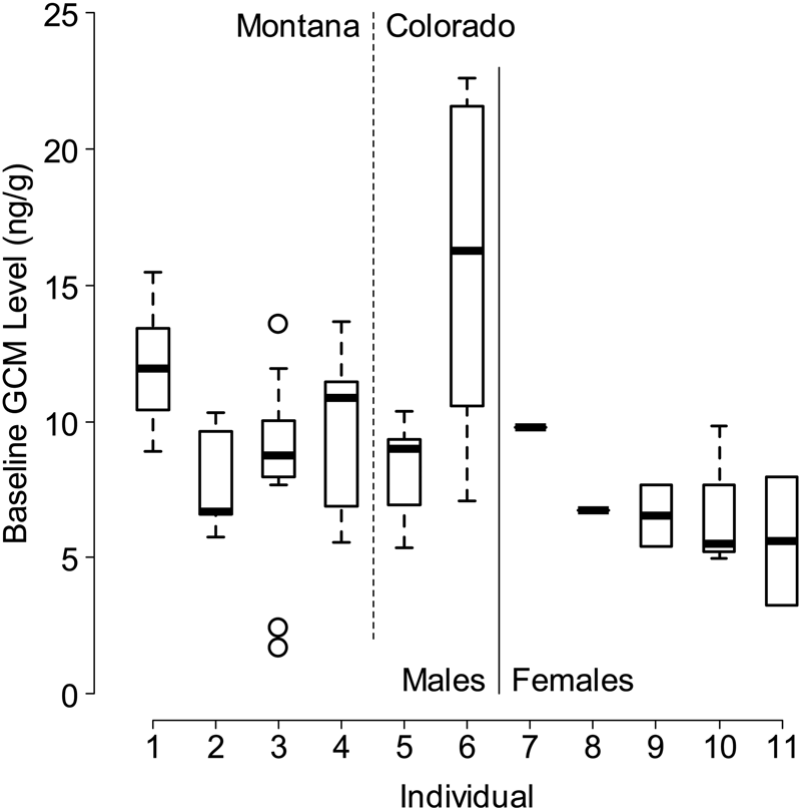
Baseline (pre-stress) levels of glucocorticoid metabolites (GCMs) measured in 11 adult American pikas (*Ochotona princeps*), including four males from Montana, two males from Colorado, and five females from Colorado. A sixth adult female sampled in Colorado did not produce faecal pellets during the pre-stress period. Sample sizes from males and females ranged from six to 18 and from one to five, respectively.

We collected 283 samples from 16 different individuals at multiple times from 0 to 28 h after capture. However, two individuals were released < 8 h after capture owing to unsafe weather conditions at the field site. Results from these individuals were not included in the final analyses, resulting in 247 samples from 14 individuals. Baseline GCM levels (Fig. 2) varied between individuals (*F* = 9.196, *P* > 0.001) and also between males and females (*F* = 22.226, *P* > 0.001), but no effect of study site location was detectable at this sample size (*t* = − 0.979 for a fixed effect of location given *n* = 60 samples from six males).

Baseline, peak, and post-peak GCM levels were higher for adult males than for females and juveniles (Table 1). Glucocorticoid metabolite levels peaked for all adult pikas 10–15 h after initiation of the stressor (capture), with the exception of one individual, in which GCM levels continued to increase after 15 h. For adults, GCM levels during the ‘peak’ period (10–15 h after capture) were 185% (±7% SEM) higher than baseline GCM levels (Table 1). Relative to samples from adults, GCM levels peaked slightly sooner (8–10 h after capture) in samples from two juveniles (one male and one female). In most cases, GCM concentration declined to nearly pre-stress levels (baseline) by the time of release (*n* = 9), although for some individuals (*n* = 3) the GCM levels remained high (Table 1). Adult males displayed a mean baseline GCM level of 10.47 ± 1.16 ng/g dry faeces and a mean peak GCM level of 19.37 ± 2.03 ng/g dry faeces. Adult females exhibited a mean GCM baseline level of 6.83 ± 0.66 ng/g dry faeces and a mean peak GCM level of 12.17 ± 1.11 ng/g dry faeces.

**Table 1.** Faecal glucocorticoid metabolites (expressed as nanograms per gram of dry faeces) measured in male and female adult and juvenile American pikas (*Ochotona princeps*)

Individual	Sex	Status	Baseline GCM	Mean peak GCM	Post-peak GCM
BYYB	M	A	11.93 ± 0.41	21.00 ± 1.46	11.95 ± 0.85
GRRY	M	A	7.62 ± 0.76	15.28 ± 0.94	13.09 ± 0.61
Mo RYYR	M	A	8.33 ± 1.07	14.33 ± 1.13	9.15 ± 0.39
BGGG	M	A	9.53 ± 1.17	22.10 ± 0.67	7.72 ± 0.58
CO RYYR	M	A	8.39 ± 0.53	15.24 ± 0.79	9.86 ± 0.41
WBWB	M	A	15.80 ± 2.23	28.27 ± 1.28	14.46 ± 0.28
YWGY	M	J	5.38 ± 0.35	7.45 ± 0.87	4.81 ± 0.31
GGBY	F	A	9.78 ± 2.65	16.92 ± 0.67	NA
YBBG	F	A	6.72 ± 0.45	11.48 ± 0.69	7.29 ± 1.08
RYYB	F	A	6.55 ± 0.58	9.42 ± 0.73	8.22 ± 0.25
BGBG	F	A	7.67 ± 1.01	13.26 ± 0.65	NA
BBGG	F	A	4.67 ± 0.23	8.76 ± 0.60	10.93 ± 0.57
GBYG	F	A	5.60 ± 1.07	13.20 ± 0.31	6.70 ± 0.25
RBGB	F	J	5.26 ± 0.11	8.39 ± 0.18	4.68 ± 0.20

Individuals are identified and named by colour-coded ear tags (e.g. BYYB), and are classified as male or female (M or F) and adult or juvenile (A or J). Values are means ± SEM. Abbreviations: GCM, glucocorticoid metabolite; and NA, not assessed.

### Biological validation of faecal glucocorticoid metabolite measurements

**Figure 3.**
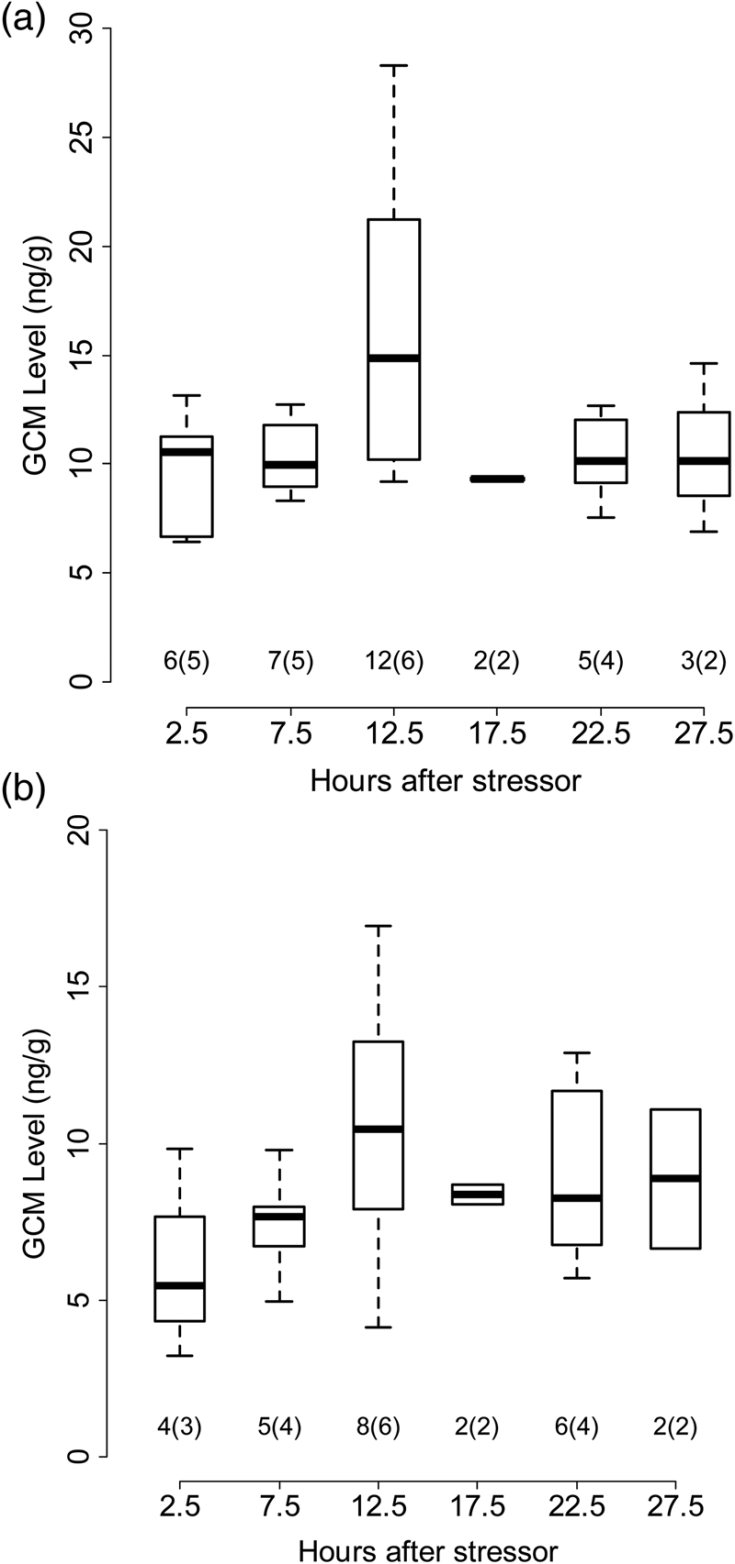
Glucocorticoid metabolite levels measured in male (**a**) and female American pikas (*Ochotona princeps*; **b**). Samples were binned into six time periods to generate summary statistics. Each box plot summarizes a median (bold line), interquartile range (box) and full range (whiskers). Sample sizes are reported as *i*(*j*), where *i* = number of faecal samples, and *j* = number of individuals producing those samples.

**Figure 4.**
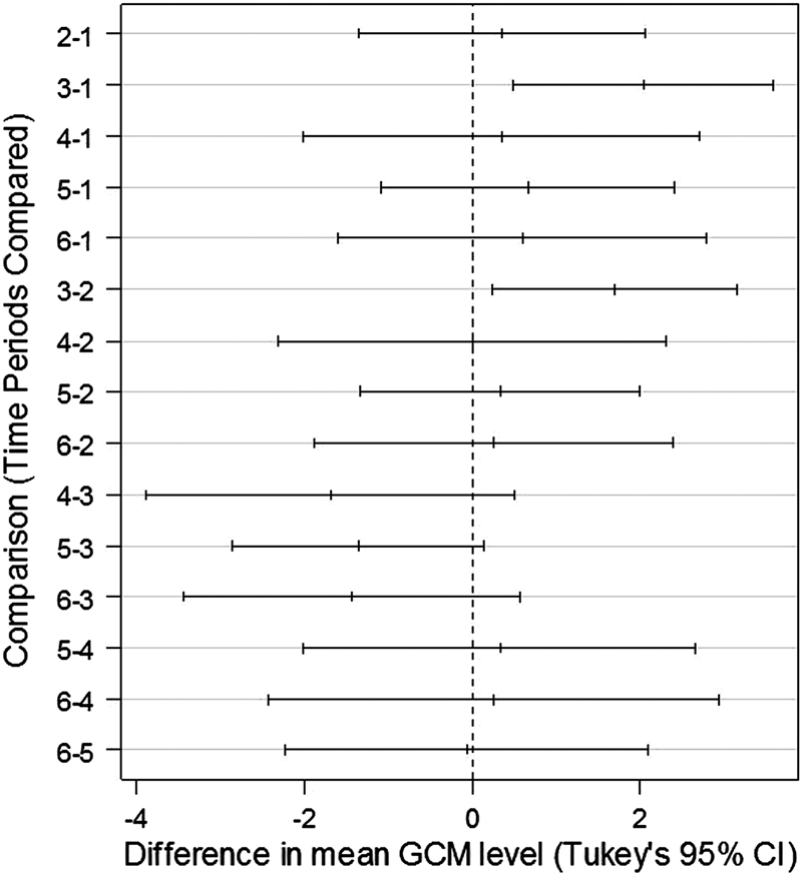
Evidence of a peak in GCM concentration during hours 10–15 (period 3 in Fig. 2) following the stress of capture and handling. Tukey's Honest Significant Difference test reveals a higher GCM level in samples from period 3 compared with periods 1 and 2 [confidence intervals (CIs) do not overlap zero], and suggests a lower GCM level in samples from periods 4–6 compared with period 3 (CIs sparely overlap zero). Standardized samples from males and females were pooled for this test (*n* = 62).

As predicted, stress related to capture and handling triggered an increase in faecal GCM level in adult male and female pikas (Fig. 3a and b). Consistent within-individual responses (Table 1) allowed us to detect an increase in GCM level subsequent to capture and restraint. Student's paired *t*-tests comparing results from *n* = 12 pikas with complete data (Table 1) revealed a significant rise in GCM level between baseline and peak periods (*t* = − 6.52, *P* < < 0.001), a significant fall in GCM level between peak and post-peak levels (*t* = 3.90, *P* = 0.001), and no significant difference between baseline and post-peak GCM levels (*t* = − 1.52, *P* = 0.07). Glucocorticoid metabolite levels clearly peaked in samples from males at 12.5 h, and peaked weakly in the same period for females (Fig. 2). Analysis of variance revealed a significant effect of time on GCM response in males only (*F* = 3.056, *P* = 0.025 for males; *F* = 1.363, *P* = 0.278 for females). However, after standardizing the response within groups (sexes) as as (where *i* = individual, *s* = sex, and *µ* and σ = mean and standard deviation in baseline GCM), we found a highly significant effect of time using combined data from males and females (*F* = 4.337, *P* = 0.002, *n* = 62). Tukey's Honest Significant Difference test confirmed that samples collected 10–15 h after capture (period 3 in Fig. 4) showed significantly higher levels of GCM than samples collected earlier.

### Timing of peak glucocorticoid metabolite level

**Figure 5.**
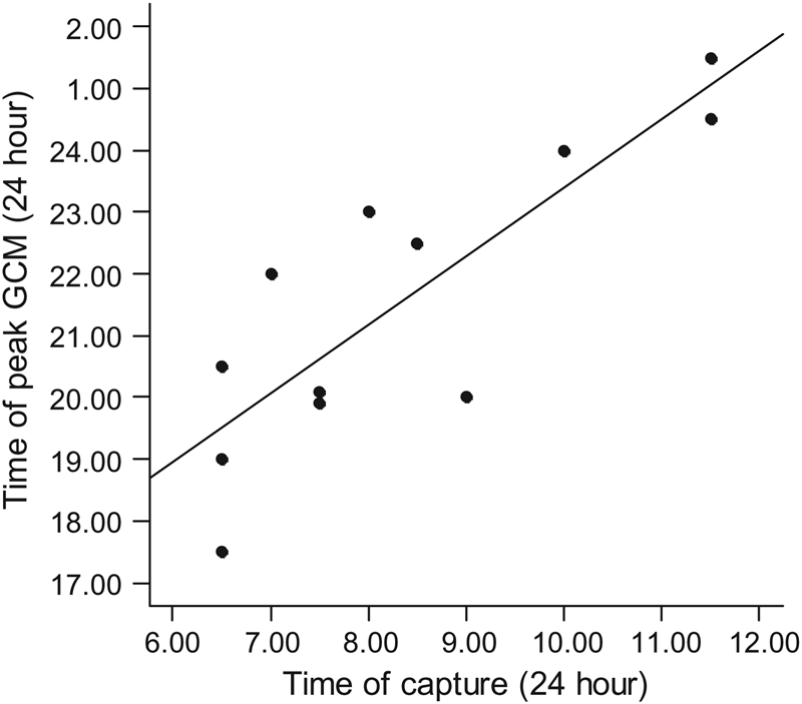
Time of peak GCM as a linear function of capture time for data from 12 American pikas (*Ochotona princeps*; adjusted *r*^2^ = 0.6633, *F* = 22.67 on 1 and 10 degrees of freedom, *P* < 0.001). Data were jittered slightly to differentiate points.

The timing of the initiation of the stressor varied among individuals, because animals entered traps at different times in the morning. All individuals were trapped between the hours of 06.30 and 11.30 h, and all pikas (except for one individual, in which GCM levels continued to climb) displayed maximal GCM levels 10–15 h after first being trapped. Individuals that were trapped later in the day had GCM levels that peaked later that evening, or early the next morning. Individuals were not synchronous in the timing of their peak GCM level; the displayed peak in GCM level was explained well by the time elapsed since the initiation of the stressor (Fig. 5) rather than circadian rhythm.

## Discussion

This study was the first to use faecal glucocorticoid metabolite analysis to assess stress in pikas. Here we recorded a stress-induced peak in GCM level that represented a 2-fold increase over baseline. A 2-fold response in GCM is relatively small in comparison to responses recorded for some species resulting from pharmacological stimulation of the stress axis through adrenocorticotrophic hormone challenge (Touma and Palme, 2005). However, a similar magnitude of response to a biological stressor has been documented for several other lagomorphs (Monclus *et al.*, 2006; Sheriff *et al.*, 2009; Scarlata *et al.*, 2011), and other small mammals (Fletcher and Boonstra, 2006; Chelini *et al.*, 2010).

Confinement and trapping has been shown repeatedly to induce a significant increase in GCM level in many mammal species (Harper and Austad, 2001; Fletcher and Boonstra, 2006; Bosson *et al.*, 2009), so we expected the stress response elicited by our methods likewise to increase GCM concentrations. Naturally occurring events that may elicit a stress response have been used for a variety of vertebrate species to validate faecal GCM analysis. Measurement of GCM levels before and after a translocation event has been used to validate faecal metabolite analysis biologically in spotted hyenas (*Crocuta crocuta*; Goymann *et al.*, 1999), dairy cattle (*Bos taurus*; Morrow *et al.*, 2002), and pygmy rabbits (*Brachylagus idahoensis*; Scarlata *et al.*, 2011). Other stressful events, such as repeated disturbance, exposure to a predator, or immobilization, have been used in biological validations for European hares (*Lepus europaeus*; Teskey-Gerstl *et al.*, 2000), snowshoe hares (*Lepus americanus*; Sheriff *et al.*, 2009), cheetahs (*Acinonyx jubatus*; Terio *et al.*, 1999), and roe deer (*Capreolus capreolu*; Dehnhard *et al.*, 2001).

The time delay between exposure to a known stressor and detection of elevated GCMs in faecal material is believed to approximate the time required for food to pass through the digestive system for a particular species (Palme *et al.*, 1996). The time delay to detection recorded here (11–15 h) is very similar to the gut passage time reported for other lagomorphs (Piekarz, 1963; Monclus *et al.*, 2006; Rehnus *et al.*, 2009; Sheriff, 2009). Thus, ∼12.5 h may be the gut passage time for American pika adults, a statistic previously unreported. We observed shorter response times for juvenile pikas, as expected if juveniles have shorter gut passage times.

As expected, measured baseline and peak GCM levels varied significantly between individuals within both male and female pikas. Numerous studies assessing adrenocortical response in vertebrate species document a large degree of variation between individuals, and this variation most probably reflects an individual's ability to cope with environmental change (Moberg, 2000; Koolhaas *et al.*, 2001). Multiple factors come into play when determining how an individual will respond physiologically to a stressor, such as previous exposure, genetics, temperament, age, and physical state (Moberg, 2000; Owen *et al.*, 2004). Additionally, sex hormones may alter the release of glucocorticoids (Von der Ohe and Servheen, 2002), and this may explain the higher overall GCM levels in males observed here. Breeding is highly seasonal in pikas, and increases in glucocorticoid level during the breeding season have been displayed in other alpine species with similarly short breeding seasons (Boonstra *et al.*, 2001). Testosterone, linked to increased aggression and conflict in males during the breeding season, has been shown to increase the release of glucocorticoids. In contrast, progesterone which is released in greater amounts in females during oestrus, pregnancy, and lactation, appears to decrease the GC response (Kenagy and Place, 2000; Owen *et al.*, 2004; Reeder *et al.*, 2004; Dantzer *et al.*, 2010). Two of the adult females used in this study were pregnant, and the remaining adult females had all recently given birth and completed lactation within the season (0–3 months prior to sampling). It is interesting to note that GCM levels in female pikas tended to remain high even after the peak GCM period, and in most cases did not decline to baseline (Table 1 and Fig. 2b). Based on our behavioural observations, adult females appeared to be more distressed in captivity, and were less active in comparison to adult males. This observation might be attributed to the physiological demands on adult females during pregnancy and lactation.

Although the secretion of glucocorticoids into the bloodstream follows a circadian rhythm (Thun *et al.*, 1981), the pattern related to GCM secretion in faecal material is less clear. Many everyday behaviours, such as arousal from sleep, are cued by the release of GCs (Krieger, 1979), and this event usually represents the largest release within a 24 h period. Thus, GC secretion typically peaks early in the morning for diurnal animals and early in the evening for nocturnal animals. Depending on the species-specific lag time required for GC metabolites to appear in faeces, one would expect a peak in GCM level in the afternoon for diurnal mammals and late at night or early the next morning for nocturnal mammals. Given that pikas are diurnal, we would have expected a peak GCM level to be recorded in the afternoon/early evening, if a circadian rhythm could be detected. However, our peak GCM levels occurred in samples collected from 17.30 to 01.30 h, and varied according to when the animal was trapped (Fig. 5). Fluctuations in daily patterns of hormone secretion are not known to vary widely among individuals within the same species (Goldman, 1999). Although some studies have detected a diurnal fluctuation in faecal GCM concentration (Bamberg *et al.*, 2001; Pihl and Hau, 2003; Bosson *et al.*, 2009), other research has documented the absence of a rhythm in faecal GCM levels (Bauer *et al.*, 2008; Chelini *et al.*, 2010). Faecal GCM concentrations reflect pooled quantities of glucocorticoids released over time, and even with frequent sampling, brief or small increases in circulating glucocorticoids could be masked by the pooling of metabolites in faeces. It may not be possible to detect diurnal changes of circulating GC levels in faecal samples for some species (Touma and Palme, 2005), especially for those that defaecate infrequently. In this study, we did not detect a circadian rhythm of GC secretion, even though we used a time series of pellets, indicating that a circadian rhythm in GCM level may not be discernible for pikas.

Pikas habitually urinate on their faecal droppings in natural environments. Current genetic and hormone research on pikas requires fresh faecal samples, and existing collection protocols specifically instruct that faecal samples can be identified as fresh only if they are still cemented to rocks with urine. Therefore, only faecal pellets coated with urine were included in our analysis, because these are the type of pellet that will be used for future studies based on non-invasive sampling.

One drawback associated with this approach is that the proportion of GCMs excreted in urine or faeces can vary based on the species. For example, research conducted on marmosets, macaques, and chimpanzees has shown that 82–91% of GCMs are excreted via the urine, while only 9–18% are excreted in faeces (Bahr *et al.*, 2000). However, for other species the opposite pattern has been displayed, with a much higher proportion of GCMs being excreted in the faeces. Following injection of radioactive corticosterone, 20% of metabolites recovered were found in the urine and 80% in the faeces of male Sprague–Dawley rats (Bamberg *et al.*, 2001). Touma *et al.* (2003) also found that radioactive metabolites were recovered predominantly in the faeces rather than in urine in mice (*Mus musculus*), and that males excreted significantly higher proportions via the faeces than females. This suggests that gender may also play a role in determining excretion patterns of metabolites, even within the same species. Given our lack of knowledge concerning the excretion route of GCMs for pikas, we cannot be certain whether our measurements more accurately reflect the GCM concentration found in urine or in faeces. However, with any particular assay, only a proportion of the total GCMs are measured, and the assay is still useful if comparisons are made using the same measured relative proportion of GCM concentration (Millspaugh and Washburn, 2004). The samples we analysed were completely saturated with urine; thus, the proportion of urine per sample did not differ among samples, eliminating this as a potential source of sample variability. By analysing faecal and urine portions together, we were able to obtain a more comprehensive estimate of total GCMs.

In conclusion, our results confirm that faecal hormone analysis can be used to assess physiological stress in the American pika; however, our approach has some limitations.

Due to field sampling constraints and the sensitivity of the species, we conducted only a biological validation of our techniques. Given the high mortality of these animals in captivity (Krear, 1965; MacArthur and Wang, 1974), information regarding optimal holding conditions is needed prior to additional laboratory-based studies. Future studies should incorporate an adrenocorticotrophic hormone challenge test or similar physiological validation, if possible (Touma and Palme, 2005). High-performance liquid chromatography could also be used to determine the exact faecal GCMs being excreted (Rose and Jusko, 1979). Furthermore, a radiolabelled metabolism study could be carried out to determine the major route and the exact rate of excretion of GCMs in pikas (Palme *et al.*, 2005). Additionally, comparisons between GCM concentration and plasma GC level measured within the same individual could serve as further validation, because GCM concentration has been positively correlated with plasma GC level for several species (Cavigelli, 1999; Mateo and Cavigelli, 2005; Sheriff *et al.*, 2010). Results from these suggested research areas would clarify information related to adrenal activity, and contribute to a better understanding of the stress response for this species.

However, current methods do provide a foundation for further research into how changes in the environment directly affect adrenocortical activity in the American pika. The methods developed and validated in the present study can be used to add non-invasive measurements of physiological stress to pika monitoring programmes and other research designed to assess pika vulnerability to predicted changes in climate. For example, by measuring GCM concentration in faecal samples collected from pikas living in different habitats, we can determine environmental correlates of GCM measurements. We caution, however, that environmental conditions (e.g. precipitation) may influence the GCM concentration measured in samples collected non-invasively (Khan *et al.*, 2002; Terio *et al.,* 2002; Millspaugh *et al.*, 2003; Palme, 2005), so that it is necessary to account for direct effects of the environment on GCM levels. For example, measured GCM concentration was higher in white-tailed deer (*Odocoileus virginianus*) faeces that had been exposed to artificial precipitation, most probably due to increased microbial activity in response to elevated moisture levels (Washburn and Millspaugh, 2002). Likewise, elevated temperatures have been shown both to increase (Millspaugh *et al.*, 2003) and to decrease (Terio *et al.,* 2002) the GCM concentration measured in faecal samples from different species. Environmental conditions may alter the GCM concentration in pika faeces, and one proposed method to test this would be to make comparisons between control samples and those exposed to different temperature and moisture levels.

Measurements of stress response have many applications in conservation. For example, Creel *et al.* (2002) compared GCM levels measured in samples collected from several national parks, in order to assess the impact of human disturbance on North American elk and wolf populations. Higher GCM levels were measured in response to increased snowmobile activity for both species (Creel *et al.*, 2002). A similar study involving maned wolves (*Chrysocyon brachyurus*) in South America found higher GCM levels from wolves living in areas outside national parks and other protected regions (Spercoski *et al.*, 2012). When using measurements of stress response to guide wildlife management decisions, it can often be difficult to separate the effects of ecological and anthropogenic disturbance on the stress response. Recent research conducted on the endangered southern resident killer whale (*Orcinus orca*) used a combination of approaches to determine the relative importance of factors affecting the population (Ayres *et al.*, 2012). Faecal thyroid and GCM measurements were used to assess the impacts of reduced prey (Chinook salmon) and increased tourist boat traffic, respectively, on whales. Results indicated that negative effects associated with boat traffic were overshadowed by a reduction in prey items, suggesting that restoration of Chinook salmon runs is most important for population recovery (Ayres *et al.*, 2012). These are a few examples of how non-invasive endocrine monitoring can help to resolve major conservation questions.

Developing stress metrics as bio-indicators is another possible conservation application. Stress in the American pika might indicate a decline in water resources key to downstream ecosystems. Pikas appear to persist primarily in locations that contain sub-surface water features, such as seasonal ice or permafrost (Millar and Westfall, 2010), and in sites with more precipitation and better winter snow cover (Beever *et al.*, 2010; Erb *et al.*, 2011), which would help to maintain sub-surface water features. These features are key to water storage and production from alpine habitats (Molotch *et al.*, 2008), and should represent an increasingly important component of water resources as surface water-storage features (glaciers and snowpacks) diminish in a warming climate (Clark *et al.*, 1994; Schrott, 1996; Millar and Westfall, 2008). Sub-surface water features also moderate the sub-surface microclimates that pikas need to survive the physiological demands of both summer and winter (MacArthur and Wang, 1973, 1974; Millar and Westfall, 2010). Thus, pikas should be stressed by a decline in the extent or quality of sub-surface water features. Such a response could serve as a bio-indicator, allowing managers to track the spatial and temporal extent of sub-surface ice features to characterize better the potential watershed productivity and ecosystem resilience. This use of stress metrics as bio-indicators of hydrological change could have several advantages over direct methods of monitoring. Monitoring a stress response may provide an early warning of ecosystem change. Using a mammalian stress response as a bio-indicator is attractive because the physical condition of a mammal represents a relatively complex set of inputs integrated over space and time. A less integrative bio-indicator might not be as effective for estimating the condition of a system that is complex and spatio-temporally extended (e.g. a watershed).
